# Music Engineering as a Novel Strategy for Enhancing Music Enjoyment in the Cochlear Implant Recipient

**DOI:** 10.1155/2015/829680

**Published:** 2015-10-12

**Authors:** Gavriel D. Kohlberg, Dean M. Mancuso, Divya A. Chari, Anil K. Lalwani

**Affiliations:** Columbia University Cochlear Implant Center, Department of Otolaryngology/Head and Neck Surgery, Columbia University College of Physicians and Surgeons, 180 Fort Washington Avenue, Harkness Pavilion 8th Floor, New York, NY 10032, USA

## Abstract

*Objective*. Enjoyment of music remains an elusive goal following cochlear implantation. We test the hypothesis that reengineering music to reduce its complexity can enhance the listening experience for the cochlear implant (CI) listener. *Methods*. Normal hearing (NH) adults (*N* = 16) and CI listeners (*N* = 9) evaluated a piece of country music on three enjoyment modalities: pleasantness, musicality, and naturalness. Participants listened to the original version along with 20 modified, less complex, versions created by including subsets of the musical instruments from the original song. NH participants listened to the segments both with and without CI simulation processing. *Results*. Compared to the original song, modified versions containing only 1–3 instruments were less enjoyable to the NH listeners but more enjoyable to the CI listeners and the NH listeners with CI simulation. Excluding vocals and including rhythmic instruments improved enjoyment for NH listeners with CI simulation but made no difference for CI listeners. *Conclusions*. Reengineering a piece of music to reduce its complexity has the potential to enhance music enjoyment for the cochlear implantee. Thus, in addition to improvements in software and hardware, engineering music specifically for the CI listener may be an alternative means to enhance their listening experience.

## 1. Introduction

The cochlear implant (CI) restores hearing to deafened individuals. Speech discrimination among the postlingually deafened CI users usually exceeds 65% [1]. Despite excellent performance on speech discrimination, enjoyment of music among CI listeners remains poor and has been attributed to decreased music perception. Music perception, the perception of pitch, melody, harmony, rhythm, and timbre, is greatly impaired in cochlear implantees [2–5]. Contributing factors responsible for diminished music perception include low resolution and skewed mapping of transmitted frequencies through the CI to the auditory cortex, difficulty perceiving spectral components individually, and deficits with higher perceptual integration tasks such as auditory stream segregation [2]. Limitations of cochlear implant hardware, sound processing software, and auditory nerve degeneration all play a possible role in signal degradation. Due to these factors, complex music signals are poorly perceived and consequently poorly enjoyed by CI listeners.

A large body of literature has shown that CI listeners have poor perception of musical elements [2–5]. In a test of 42 CI users and 10 normal hearing (NH) adults, CI users were found to be significantly worse than NH adults at pitch perception as well as both melody and timbre recognition [5]. In a study of 9 adults who underwent cochlear implantation, pitch perception was found to be worse after implantation than immediately before implantation [3].

Enjoyment of music has also been shown to decrease following cochlear implantation. In approaching the issue of poor musical enjoyment in CI users, studies have taken two main approaches. In the first, attempts have been made to analyze how varying CI devices and strategies affect music enjoyment. In the second, CI users' music enjoyment has been analyzed across different genres and pieces of music with varying complexities. In most studies, music enjoyment has been assessed by variations of a bipolar visual analog scale (VAS) or a discrete 10-point scale [6–9]. Questionnaire studies of CI users have found a significant decrease in music enjoyment as well as time devoted to listening to music compared with before onset of deafness. Many CI listeners could not enjoy music at all, stating that music did not sound natural. Despite the overall decrease in enjoyment, 38–73.6% of CI users still listened to music and 30.2%–37% stated they would undergo implantation simply to be able to listen to music [10–13].

Attempts to improve music enjoyment have examined cochlear implant hardware and software strategies with mixed results [14]. A study in CI users with a MED-EL device on one side and a cochlear nucleus on the other found no significant difference in music enjoyment when either device type was used exclusively [6]. Similarly, two other studies comparing fine structure processing (FSP) strategy to continuous interleaved sampling (CIS) strategy found no difference in music enjoyment among CI listeners [8, 15].

Given the significant reduction in the population of auditory neurons available to relay complex musical signals in severe to profoundly deafened patients undergoing implantation, it may be unrealistic to expect normal music perception despite further enhancement in CI software and hardware. In the absence of significant improvement in perception, music may have to be specially engineered for the CI listener to enhance its enjoyment. In exploration of this concept, we investigate the features of music that impact on its enjoyment (not perception) in normal hearing individuals and cochlear implantees.

Music perception among CI listeners has been studied extensively [3–5]. Investigating perception of music among implantees is important in assessing the ability of CI software and hardware to restore musical perception and may prove beneficial in guiding our understanding of music enjoyment among CI listeners. It is imperative to note, however, that music perception and music enjoyment are fundamentally different and may not necessarily correlate. For example, Alexander et al. in their study of music perception and enjoyment found that, despite significantly poorer performance on music perception, the enjoyment of music among cochlear implantees was comparable to normal hearing individuals [16]. This disconnect between enjoyment and perception is similar to a cochlear implantee performing well on speech perception testing but finding the quality of speech sound unpleasant or unnatural. Thus, music perception cannot be substituted for music enjoyment when investigating the types and characteristics of music that are enjoyable to CI listeners; consequently, the enjoyment of music must be studied directly.

## 2. Materials and Methods

### 2.1. Participants

After obtaining Columbia University Medical Center Institutional Review Board approval, we analyzed music enjoyment in 16 normal hearing (NH) individuals and 9 cochlear implantees. After obtaining consent, NH subjects underwent an audiologic evaluation, including evaluation of pure tone thresholds, speech discrimination, and otoscopic evaluation. Inclusion criteria included English speaking, 18 years of age or older, no history of hearing loss, and pure tone audiometric thresholds less than or equal to 20-decibel hearing loss in both ears at all tested frequencies. Inclusion criteria for cochlear implantees included English speaking, age over 18 years, and postlingual deafness.

### 2.2. Music Piece

Each subject listened to and evaluated an original and 20 modified versions of a 20-second piece of country music (“Milk Cow Blues” performed by Angela Thomas Wade) recorded in multitrack format. The original song segment included multiple musical elements: female vocals; three melodic instruments (guitar, piano, and fiddle); rhythmic drums including the snare. Each of the 20 modified versions was comprised of a unique subset of musical elements of the original song. Five modified versions were comprised of a single musical element: vocals; piano; guitar; fiddle; and the snare. Seven modified versions were obtained by combining two musical elements: vocals and guitar; vocals and snare; vocals and piano; vocals and fiddle; snare and guitar; snare and piano; and snare and fiddle. Four modified versions were obtained by combining three musical elements: vocals, snare, and guitar; vocals, snare, and piano; vocals, snare, and fiddle; guitar, piano, and fiddle. Two modified versions were comprised of four musical elements: piano, guitar, fiddle, and vocals; snare, kick, overhead, and tom drums. One version was comprised of five musical elements: snare, kick, overhead, tom drums, and vocals. One version with all of the musical elements except for the vocals contained 10 musical elements.

### 2.3. Music Presentation to NH Listeners

The subjects listened to the musical samples in a sound proof booth (IAC Acoustics, New York, NY). Participants adjusted the volume according to their preference. Participants rated each of the music segments using a visual analog scale implemented using MATLAB version 7.1 (Mathworks, Natick, MA) on a MacBook Air (Apple, Cupertino, CA). Subjects were instructed to rate each sample on a scale from 0 to 10 in each of the following categories: “pleasant and unpleasant,” “sounds like music and does not sound like music,” and “natural and unnatural,” with higher numeric scores corresponding to higher levels of pleasantness, musicality, and naturalness. Subjects were presented the music samples in a random order.

Each of the music segments was then processed through CI simulation software provided by Advanced Bionics Corp., using MATLAB version 7.1 [17]. These music segments with CI simulation were then presented and rated by the NH listeners in the same fashion as the music segments without CI simulation.

### 2.4. Music Presentation to CI Listeners

Listening took place in a sound proof booth. Music segments were presented in a free field at 60 dB SPL. Participants rated each music segment in a similar fashion as the NH listeners as described above.

### 2.5. Cochlear Implant Simulation Software

Half of the music segments were presented to the NH listeners after being processed through a CI simulation. The CI simulation software aimed to simulate a CI listening experience by modeling both the sound processing that occurs to an incoming sound signal in the speech processor of the cochlear implant and the spread of excitation related to electrical stimulation in the cochlea [17]. In particular, CI sound processing was modeled by filtering the sound signal into 15 logarithmically spaced channels covering the range from 350 to 5500 Hz. The envelope signal was computed for each channel and used to modulate a noise band. The noise band for each channel was chosen to have the center frequency corresponding to the center frequency of the channel and to simulate appropriate spread in the cochlea. Litvak et al. [17] varied the amount of spread in the noise band in dB/octave and determined that 20 dB/octave appeared as an accurate model of spread that occurs for the average CI listener. In addition, they showed that this simulation matched NH listeners' speech discrimination on vowel recognition.

### 2.6. Statistical Analysis

*t*-tests were used to compare enjoyment between subsets of musical segments within the NH listeners and the CI listeners. In addition, analysis of variance (ANOVA) was applied to compare the mean enjoyment of the original version of the song and the mean enjoyment of modified versions comprised of 1, 2, or 3 instruments. Enjoyment comparisons were analyzed separately for pleasantness, musicality, and naturalness scales. Statistical significance was considered for *P* < 0.05 for both the *t*-tests and ANOVA statistical tests. Of note, all 4 of the modified versions of the song with 4 or more musical elements were excluded in the linear regression results. Three of these modified versions contained percussion elements that were not present in any of the other modified versions, compromising the generalizability of any results related to these modified versions. The fourth excluded modified version was the only modified version remaining with 4 musical elements, limiting the relevance of this category. Of note, all statistically significant linear regression findings hold when including these 4 excluded modified versions.

## 3. Results

The mean age of the 16 NH participants was 29 years (25–33). Eight were female and eight were male. Eighty-eight percent spoke English as their first language. Speech discrimination was 96–100% on 25 spondee words. One participant had a history of pressure equalization tube placement as a child.

The mean age of the cochlear implantees was 54 years (26–74). Six were female and 3 were male. One hundred percent spoke English as their first language. Six were implanted with the Advanced Bionics HiRes 90K implant and 3 with the Advanced Bionics CII implant. All CI listeners used the Fidelity 120 sound processing strategy. The average time since implantation was 7 years (2–13). Three had bilateral cochlear implants. Speech discrimination ranged from 60 to 97% (mean 84.6%) on the AzBio Sentence list.

### 3.1. Enjoyment of the Original Music Sample

The original version of the song “Milk Cow Blues” was well liked by the NH listeners without CI simulation (pleasant, musical, and natural; 8.3, 8.8, and 8.4). NH listeners rated the original version of the song as the 3rd most pleasant, 2nd most musical, and 5th most natural out of the 21 total segments (original plus 20 modified versions). There was no modified version of the song that was preferred by the NH listeners on all three enjoyment scales (pleasantness, musicality, and naturalness). With CI simulation, NH listeners rated the original version poorly (pleasant, musical, and natural; 0.69, 1.4, and 0.76) and rated the original version 16th most pleasant, 13th most musical, and 12th most natural. Ten of the 20 modified versions were preferred to the original on all three enjoyment scales by the NH listeners with CI simulation. The CI listeners rated the original version of the song very poorly (pleasant, musical, and natural: 5.4, 6.3, and 5.6) compared to the modified versions of the song ranking it as the least pleasant and the 2nd least musical and natural. CI listeners preferred 19 of the 20 modified versions to the original on all three enjoyment scales.

### 3.2. Comparison of the Original Music Sample to Modified Version with 1–3 Instruments

#### 3.2.1. Preferences among NH Listeners without CI Simulation

**Figure 1:**
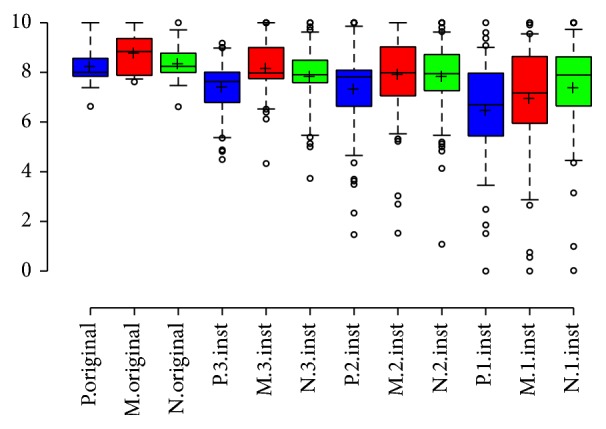
NH listeners without CI simulation enjoyment by number of instruments. NH listeners preferred the original music sample compared to modified segments comprised of a single instrument, two instruments, or three instruments. P: pleasant, N: natural, M: sounds like music, and Inst: instruments. Center lines show the medians, box limits indicate the 25th and 75th percentiles, whiskers extend to 5th and 95th percentiles, outliers are represented by dots, and crosses represent sample means.

Music enjoyment was universally greater for NH listeners for the original music segment compared to modified versions comprised of a single instrument, two instruments, or three instruments (Figure 1). Compared to the original sample, there was a relative reduction in pleasantness of 21.4%, 9.0%, and 9.5% for modified versions with a single instrument, two instruments, and three instruments, respectively (*P* < 0.05). The relative reduction in enjoyment of the original music sample compared to modified versions with 1–3 instruments for the three scales (pleasant, musical, and natural) is summarized in Table 1. A linear regression analysis of enjoyment with ANOVA found a significant difference in the means for the original version and for modified versions with 1–3 instruments (*P* < 0.01 for pleasant, musical, and natural scales).

#### 3.2.2. Preferences among NH Listeners with CI Simulation

**Figure 2:**
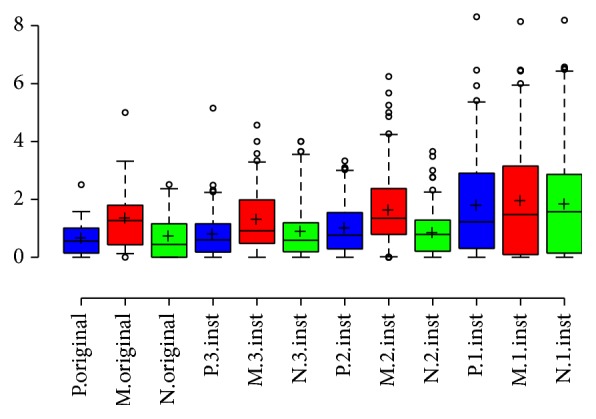
NH listeners with CI simulation enjoyment by number of instruments. NH listeners preferred modified segments comprised of a single instrument, two instruments, or three instruments compared to the original music sample. P: pleasant, N: natural, M: sounds like music, and Inst: instruments. Center lines show the medians, box limits indicate the 25th and 75th percentiles, whiskers extend to 5th and 95th percentiles, outliers are represented by dots, and crosses represent sample means.

When listening with CI simulation, NH listeners rated the original music segment less enjoyable than modified versions comprised of one to three musical elements (Figure 2). Compared to the original music sample, modified versions with a single instrument were significantly more pleasant by 59.5% (*P* < 0.05). The relative increase in enjoyment for the modified versions with 1–3 instruments compared to the original song for NH listeners with CI simulation is summarized in Table 2. A linear regression analysis of enjoyment with ANOVA found a significant difference in the means for the original version and for modified versions with 1–3 instruments on the pleasant scale (*P* = 0.001) and natural scale (*P* = 0.003) but not the musical scale (*P* = 0.09).

#### 3.2.3. Preferences among CI Listeners

**Figure 3:**
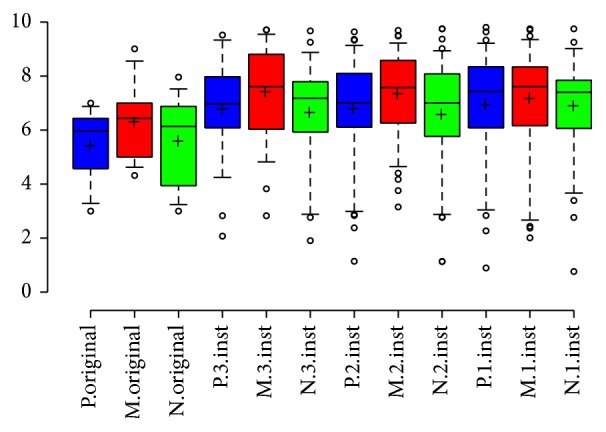
CI listeners enjoyment by number of instruments. CI listeners preferred modified segments comprised of a single instrument, two instruments, or three instruments compared to the original music sample. P: pleasant, N: natural, M: sounds like music, and Inst: instruments. Center lines show the medians, box limits indicate the 25th and 75th percentiles, whiskers extend to 5th and 95th percentiles, outliers are represented by dots, and crosses represent sample means.

CI listeners rated the original music segment less enjoyable than modified versions comprised of one to three musical elements (Figure 3). Compared to the original music sample, modified versions with a single instrument and 3 instruments were significantly more pleasant by 21.9% and 18.3%, respectively (*P* < 0.05). The relative increase in enjoyment for the modified versions with 1–3 instruments compared to the original song for NH listeners with CI simulation is summarized in Table 3. A linear regression analysis of enjoyment with ANOVA found a significant difference in the means for the original version and for modified versions with 1–3 instruments on the pleasant scale (*P* = 0.034) but not the musical (*P* = 0.22) or natural scales (*P* = 0.21).

### 3.3. Comparison of Modified Versions with and without Vocals

The original music sample contained prominent female vocals. Modified versions (*n* = 10) containing the vocal element of the original music sample were compared to modified versions (*n* = 10) that did not include the vocals. NH listeners without CI simulation found versions with the vocals significantly more enjoyable on all three scales, pleasant, musical, and natural (*P* < 0.01). In contrast, with CI simulation, NH listeners found versions with vocals significantly less enjoyable on all three scales (*P* < 0.02). For the CI listeners, there was virtually no difference in enjoyment between versions with and without vocals (mean pleasant, musical, and natural with and without vocals: 6.84 versus 6.85, 7.24 versus 7.44, and 6.66 versus 6.76, resp.).

### 3.4. Comparison of Modified Versions with Melodic Elements with and without Rhythmic Elements

Modified versions of the original song with melodic elements only (vocals, piano, fiddle, and guitar) were compared to versions that also included rhythmic drum instruments. All modified versions of the song were used in this comparison except for the version with only the snare and the version with only the four drum instruments, as these segments did not include any melodic elements. There was no difference in enjoyment on all three scales for NH listeners without CI simulation between versions with and without rhythmic instruments. NH listeners with CI simulation found the rhythmic versions significantly more enjoyable on all three scales (*P* < 0.05 for pleasant, musical, and natural). CI listeners found virtually no difference in enjoyment between versions with and without rhythmic instruments (mean pleasant, musical, and natural with and without rhythmic instruments: 6.80 versus 6.89, 7.21 versus 7.40, and 6.73 versus 6.70, resp.).

## 4. Discussion

In this study, we investigated features of music that impact on its enjoyment and tested the novel hypothesis that an original piece of music could be reengineered to make it more enjoyable for the cochlear implantee. To the best of our knowledge, no prior study has taken the approach of altering a specific piece of music to determine if it can be made more enjoyable for CI listeners. We modified a 20-second segment of a country music song by playing it with various subsets of the original music sample's musical elements. Modified versions contained various combinations of the vocals and instruments.

We found that NH listeners enjoyed the original music segment the most and rated the modified, less complex, versions less enjoyable. On the other hand, for the NH listeners with CI simulation, enjoyment increased significantly when the complexity of the original musical sample was reduced by limiting the number of elements in modified versions of the song to 1–3 elements. The extent of increase in enjoyment of these less musically complex modified music segments was even more pronounced among the CI listeners.

Studies on music perception have shown that CI listeners have severe difficulty identifying pitch, timbre, and melody, the main qualities in vocals and melodic instruments such as guitar, piano, and fiddle [3, 5]. On the other hand, the musical quality best perceived by CI listeners has been found to be rhythm [4]. In our study we found that NH listeners with CI simulation preferred modified music samples that included rhythmic instruments compared to segments containing only melodic instruments. Interestingly, the CI listeners did not rate samples with and without rhythmic instruments differently. The impact of rhythmic instruments will need to be further studied with musical pieces containing greater numbers of rhythmic instruments.

The effect of vocals on music perception has been studied in CI listeners. A study of 87 CI listeners found that the presence of lyrics in pop and country songs significantly improves the CI listeners' ability to identify a melody [18]. In this study, we evaluated the effect of vocals on music enjoyment. We found that while enjoyment was greater for music with vocals for NH listeners without CI, it was significantly less for NH listeners with CI. CI listeners, on the other hand, rated modified segments with and without vocals exactly the same.

We found that for NH listeners with CI simulation enjoyment was significantly increased for modified segments without vocals and for modified segments with rhythmic instruments. While we did not find that music segments limited to particular qualities, such as only vocals, melodic, or rhythmic elements, were significantly more enjoyable to CI listeners, we did find that reducing the number of musical elements significantly increased music enjoyment both for the NH listeners through CI simulation and for the CI listeners.

Several studies have examined the relationship between music enjoyment and complexity of musical pieces in CI listeners. In a study of 15 CI users and 24 hearing aid (HA) users meeting audiologic criteria for CI, they were asked to rate their enjoyment of music played by a single instrument, solo instruments with background accompaniment, and ensembles. The study found that music played by a single instrument was more enjoyable than music played by multiple instruments to the CI and HA listeners when analyzed together. Of note, the music played by the single instrument, solo instruments with background accompaniment, and ensembles was not specified to be the same music segment [7]. A study of 20 NH listeners with CI simulation processing found that minimizing reverberation time increased music enjoyment [19].

Part of this study involved querying normal hearing listeners' enjoyment of music through a CI simulation validated for speech perception. There are limitations to using CI simulation as a proxy for CI listeners' enjoyment. In our study, relative enjoyment of modified music segments was different between the NH listeners with CI simulation and the CI listeners. A previous study with CI simulation by Wright and Uchanski also found significant differences between music perception and enjoyment in NH listeners with CI simulation and CI listeners [20]. While the study by Wright and Uchanski and this study did not show clear correspondence in music enjoyment between NH listeners with CI and CI listeners, there is reason to continue to attempt simulation studies. There are advantages to conducting initial or exploratory studies with CI simulation in NH listeners. The CI listener population has significant variation in age, duration of deafness, etiology of deafness, musical background and training, and rehabilitative outcomes. The use of NH listeners of similar age, hearing and musical training avoids these limitations typical of implanted population. Continued study of NH listeners with CI simulation processing may lead to an accurate prediction of CI listener enjoyment, which would be of great benefit.

In this study only a single piece of music was studied. Further analysis of other genres of music and other pieces within the country music genre will need to be examined. Additionally, other methods for altering the complexity of music beyond including and excluding vocals and instruments need to be explored.

## 5. Conclusion

Musical enjoyment with or without CI is influenced by the complexity of the original music. Our study offers preliminary evidence that engineering of music to reduce the complexity of music has the potential to make listening more enjoyable for the CI listener. Thus, in addition to improvement in software and hardware, engineering of music specifically for a CI listener may be an important way to enhance his or her listening experience.

## Figures and Tables

**Table 1:** Relative reduction of enjoyment of the original music sample compared to modified versions containing 1–3 instruments among NH listeners without CI simulation.

Relative reduction in enjoyment	Original versus 1 instrument	Original versus 2 instruments	Original versus 3 instruments
Pleasant	**21.4**	**9**	**9.5**
Musical	**18.5**	**7.5**	**7.2**
Natural	**11.6**	**6.1**	6.2

Values in bold are for *P* < 0.05.

**Table 2:** Relative increase of enjoyment of the original music sample compared to modified versions containing 1–3 instruments among NH listeners with CI simulation.

Relative increase in enjoyment	Original versus 1 instrument	Original versus 2 instruments	Original versus 3 instruments
Pleasant	**59.5**	33	**15.6**
Musical	15.1	14.4	88.7
Natural	51.7	7.9	15

Values in bold are for *P* < 0.05.

**Table 3:** Relative increase of enjoyment of the original music sample compared to modified versions containing 1–3 instruments among CI listeners.

Relative increase in enjoyment	Original versus 1 instrument	Original versus 2 instruments	Original versus 3 instruments
Pleasant	**21.9**	20.1	**18.3**
Musical	**12.2**	14.1	13.5
Natural	**17.8**	15	15.3

Values in bold are for *P* < 0.05.

## References

[1] Holden L. K., Finley C. C., Firszt J. B. (2013). Factors affecting open-set word recognition in adults with cochlear implants. *Ear and Hearing*.

[2] Limb C. J., Roy A. T. (2014). Technological, biological, and acoustical constraints to music perception in cochlear implant users. *Hearing Research*.

[3] Looi V., McDermott H., McKay C., Hickson L. (2008). The effect of cochlear implantation on music perception by adults with usable pre-operative acoustic hearing. *International Journal of Audiology*.

[4] Kong Y.-Y., Cruz R., Jones J. A., Zeng F.-G. (2004). Music perception with temporal cues in acoustic and electric hearing. *Ear and Hearing*.

[5] Kang R., Nimmons G. L., Drennan W. (2009). Development and validation of the University of Washington clinical assessment of music perception test. *Ear and Hearing*.

[6] Harris R. L., Gibson W. P. R., Johnson M., Brew J., Bray M., Psarros C. (2011). Intra-individual assessment of speech and music perception in cochlear implant users with contralateral Cochlear and MED-EL systems. *Acta Oto-Laryngologica*.

[7] Looi V., McDermott H., McKay C., Hickson L. (2007). Comparisons of quality ratings for music by cochlear implant and hearing aid users. *Ear and Hearing*.

[8] Looi V., Winter P., Anderson I., Sucher C. (2011). A music quality rating test battery for cochlear implant users to compare the FSP and HDCIS strategies for music appreciation. *International Journal of Audiology*.

[9] Rosslau K., Spreckelmeyer K. N., Saalfeld H., Westhofen M. (2012). Emotional and analytic music perception in cochlear implant users after optimizing the speech processor. *Acta Oto-Laryngologica*.

[10] Philips B., Vinck B., De Vel E. (2012). Characteristics and determinants of music appreciation in adult CI users. *European Archives of Oto-Rhino-Laryngology*.

[11] Lassaletta L., Castro A., Bastarrica M. (2007). Does music perception have an impact on quality of life following cochlear implantation?. *Acta Oto-Laryngologica*.

[12] Lassaletta L., Castro A., Bastarrica M. (2008). Changes in listening habits and quality of musical sound after cochlear implantation. *Otolaryngology—Head and Neck Surgery*.

[13] Kohlberg G., Spitzer J. B., Mancuso D., Lalwani A. K. (2014). Does cochlear implantation restore music appreciation?. *The Laryngoscope*.

[14] Drennan W. R., Rubinstein J. T. (2008). Music perception in cochlear implant users and its relationship with psychophysical capabilities. *Journal of Rehabilitation Research and Development*.

[15] Magnusson L. (2011). Comparison of the fine structure processing (FSP) strategy and the CIS strategy used in the MED-EL cochlear implant system: speech intelligibility and music sound quality. *International Journal of Audiology*.

[16] Alexander A. J., Bartel L., Friesen L., Shipp D., Chen J. (2011). From fragments to the whole: a comparison between cochlear implant users and normal-hearing listeners in music perception and enjoyment. *Journal of Otolaryngology—Head & Neck Surgery*.

[17] Litvak L. M., Spahr A. J., Saoji A. A., Fridman G. Y. (2007). Relationship between perception of spectral ripple and speech recognition in cochlear implant and vocoder listeners.

[18] Gfeller K., Jiang D., Oleson J. J. (2012). The effects of musical and linguistic components in recognition of real-world musical excerpts by cochlear implant recipients and normal-hearing adults. *Journal of Music Therapy*.

[19] Certo M. V., Kohlberg G. D., Chari D. A., Mancuso D. M., Lalwani A. K. (2010). Reverberation time influences musical enjoyment with cochlear implants. *Otology & neurotology*.

[20] Wright R., Uchanski R. M. (2012). Music perception and appraisal: cochlear implant users and simulated cochlear implant listening. *Journal of the American Academy of Audiology*.

